# Deep resequencing reveals allelic variation in *Sesamum indicum*

**DOI:** 10.1186/s12870-014-0225-3

**Published:** 2014-08-20

**Authors:** Linhai Wang, Xuelian Han, Yanxin Zhang, Donghua Li, Xin Wei, Xia Ding, Xiurong Zhang

**Affiliations:** Oil Crops Research Institute of the Chinese Academy of Agricultural Sciences, Key Laboratory of Biology and Genetic Improvement of Oil Crops of the Ministry of Agriculture, Wuhan, 430062 China; Beijing Genomics Institute (BGI) − Shenzhen, Shenzhen, China; 1gene, Hangzhou, China

**Keywords:** *Sesamum indicum*, Resequencing, Variation, Linkage disequilibrium

## Abstract

**Background:**

Characterization of genome-wide patterns of allelic variation and linkage disequilibrium can be used to detect reliable phenotype–genotype associations and signatures of molecular selection. However, the use of *Sesamum indicum* germplasm for breeding is limited by the lack of polymorphism data.

**Results:**

Here we describe the massively parallel resequencing of 29 sesame strains from 12 countries at a depth of ≥ 13-fold coverage for each of the samples tested. We detected an average of 127,347 SNPs, 17,961 small InDels, and 9,266 structural variants per sample. The population SNP rate, population diversity (π) and Watterson’s estimator of segregating sites (θw) were estimated at 8.6 × 10^-3^, 2.5 × 10^-3^ and 3.0 × 10^-3^ bp^-1^, respectively. Of these SNPs, 23.2% were located within coding regions. Polymorphism patterns were nonrandom among gene families, with genes mediating interactions with the biotic or abiotic environment exhibiting high levels of polymorphism. The linkage disequilibrium (LD) decay distance was estimated at 150 kb, with no distinct structure observed in the population. Phylogenetic relationships between each of the 29 sesame strains were consistent with the hypothesis of sesame originating on the Indian subcontinent. In addition, we proposed novel roles for adenylate isopentenyltransferase (ITP) genes in determining the number of flowers per leaf axil of sesame by mediating zeatin biosynthesis.

**Conclusions:**

This study represents the first report of genome-wide patterns of genetic variation in sesame. The high LD distance and abundant polymorphisms described here increase our understanding of the forces shaping population-wide sequence variation in sesame and will be a valuable resource for future gene–phenotype and genome-wide association studies (GWAS).

**Electronic supplementary material:**

The online version of this article (doi:10.1186/s12870-014-0225-3) contains supplementary material, which is available to authorized users.

## Background

*Sesamum indicum* (sesame) is an ancient crop with a mid-range genome size of ~357 Mb, and contains high concentrations of oils and proteins with medicinal value. However, this species is prone to waterlogging, and is particularly susceptible to many fungal and bacterial diseases, such as stem and root rot, *Fusarium* wilt, powdery mildew and others. These biotic and abiotic stresses can lead to lower overall yields, with outputs strongly associated with growth conditions. To overcome environmental stresses and improve yields, abundant germplasm along with genetic information are required for plant-breeding programs [1], and characterization of genome-wide patterns of allele variation and linkage disequilibrium ensure the detection of reliable phenotype–genotype associations and signatures of molecular selection [2]. India, China and Korea are the leading countries for sesame germplasm collection, preservation and research [3]. In China, ~6,000 strains of sesame have been deposited in the National Gene Bank of China (Wuhan, medium-term Genebank; Beijing, long-term Genebank). In Korea, > 7,698 variants have been preserved in the Gene Bank of the Rural Development Administration (RDA) located in Suwon, Korea [4], and in India > 10,000 variants have been archived in the National Bureau of Plant Genetic Resources (New Delhi, India). However, few studies have examined the genetic diversity of the sesame germplasm on a genome-wide scale due to a lack of genomic information and an absence of suitable biomarkers [1,5-7].

Sesame is the most common cultivar of the genus *Sesamum*, which contains more than 20 species of flowering plants. Unlike sesame, the majority of species in this genus have not been domesticated, with significant divergence in polyploidy levels [1]. As most of these wild species are native to sub-Saharan Africa, sesame was originally believed to have originated in Africa; however, domesticated sesame has since been shown to have originated on the Indian subcontinent [8,9]. Further investigation into the evolution of sesame has been hampered by the absence of detailed molecular data across multiple sesame strains.

Completion of the sesame reference genome provides an essential resource for exploring the genetic variation of wild and domesticated *S. indicum* (http://www.ocri-genomics.org/Sinbase/). Here, we analyzed 29 resequenced sesame strains collected from 12 countries at a coverage of ≥ 13-fold. From these data, we have constructed the first haplotype map for sesame, which provides insight into the genetic diversity of sesame across multiple strains. These data can be used for the development of genome-wide association studies, and in turn facilitate the mapping of genes associated with both simple and complex traits.

## Results and discussion

### Phenotype diversity of resequenced sesame strains

**Figure 1:**
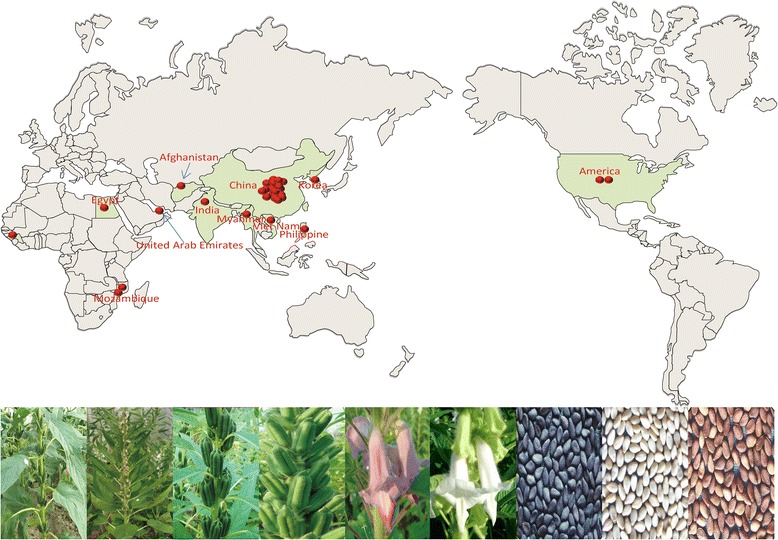
**Origins of the sesame strains used in this study.**

We manually selected 29 sesame strains for genome resequencing, including 6 from its presumed origin sites of India and Africa, 16 from China, 2 from the United States and 1 each from Afghanistan, the United Arab Emirates, Korea, Myanmar, the Philippines and Viet Nam (Additional file 1: Data S1). These strains exhibited a wide range of phenotypes, including determinate and indeterminate growth habits, tall and short plant height, early and late flowering, different seed coat color, single and triple flowers per leaf axil, uniculm and branching style, and others. The distant geographic relationships and wide phenotype variation made these strains an ideal model for exploration of the genetic diversity of cultivated sesame (Figure 1).

### Landscape of the genetic diversity of sesame

To identify large-scale polymorphisms and better understand the genetic structure of the sesame germplasm, each of the 29 sesame strains were re-sequenced, generating more than 120 Gb of filtered data at a coverage depth of ≥ 13× for each strain (Additional file 2: Figure S1; Additional file 1: Data S1). All sequence reads were aligned against the reference genome of “Zhongzi No. 13”, which has an effective genome length of 274 Mb (http://www.ocri-genomics.org/Sinbase/), using the BWA software [10]. The mapping rate across different strains varied from 88.8% to 95.2%, for an average of 91.4%. The mapping result is consistent with that from the GATK software (Additional file 1: Data S1).

Using a stringent pipeline, we identified an average of 127,347 single nucleotide polymorphisms (SNPs) per strain using the SAMtools software [11], ranging from 40,925 to 392,544 (Table 1; Additional file 2: Figure S2). Overall, SNP rates ranged from 1.5 × 10^-4^ to 14.3 × 10^-4^, respectively, with G:A, A:G, C:T and T:C substitutions being the most common (Additional file 2: Figure S3). By combining SNP across all strains, we identified 2,348,008 unique SNPs, for a population SNP rate of 8.6 × 10^-3^ bp^-1^. We next employed GATK software to call and calculate the SNP population, which resulted in generation of a total of 2,003,821 population SNPs. The concordance rates between GATK and SAMtools ranged from 80.0% to 89.9% with an average of 85.4% for the 16 linkage groups (pseudomolecular chromosomes) (Additional file 1: Data S2). Sanger sequencing showed that the SNP calling accuracy rate was ~93.7% (Additional file 2: Figure S4; Additional file 1: Data S3). These results indicated that the majority of SNPs detected here were reliable. Of these SNPs, 25.1% were located within gene coding regions with 1.5%, 7.5% and 1.7% in the 5′ untranslated region (UTR), coding sequence (CDS) and 3′UTR, respectively (Additional file 2: Figure S5; Additional file 1: Data S4). The top three SNP rates were detected in strains 20, 24 and 26, which originated in India, Myanmar and the United Arab Emirates, respectively; thus these regions may harbor a more diverse sesame germplasm.

**Table 1:** **Summary of DNA variations in the 29 sesame strains**

**No.**	**Strain name**	**Total bases (Gb)**	**Total SNPs (number)**	**Total SNPs in gene (%)**	**Total InDels (number)**	**Total InDels in gene (%)**
1_CHN	Zhongzhi No.15	4.21	73,716	28.5	11,441	28.6
2_CHN	Zhongzhi No.11	4.22	52,471	17.1	4,495	27.9
3_CHN	H98	4.05	123,830	22.8	17,998	27.4
4_CHN	Jizhi No.1	4.18	107,656	21.4	9,931	24.9
5_CHN	Jinzhi No.2	4.09	92,668	20.2	5,966	25.6
6_CHN	Zhima8131	3.81	109,905	37.1	25,752	33.9
7_CHN	2009-3335_2	4.06	208,773	27.8	37,602	28.3
8_CHN	ZZM2541	4.12	62,528	25.3	8,128	32.4
9_CHN	Yiyangbai	4.22	68,640	24.2	11,851	23.5
10_CHN	Zihuaye 23	4.20	46,507	17.8	7,289	24.3
11_CHN	Baizhima	4.21	176,649	25.4	32,028	26.9
12_CHN	Zhima	4.17	68,617	20.7	11,531	25.5
13_CHN	Mishuozhima	4.17	89,048	21.1	14,073	25.1
14_CHN	Bahuama	4.21	160,011	32.3	32,041	28.9
15_CHN	Xiangheizhi 2078	4.17	92,303	19.1	8,676	21.8
16_CHN	Fuyangsilengcao	4.07	79,310	16.8	9,436	26.5
17_AFG	0725	4.10	136,555	29.2	21,952	34.7
18_EGY	L161	4.15	203,642	27.6	26,351	28.9
19_GUI	K1	4.12	137,736	20.0	12,656	26.4
20_IND	0847	4.15	392,544	30.9	56,594	30.0
21_KOR	Shuiyuan 117	4.19	63,828	17.4	8,815	22.4
22_MOZ	Suke No5- < 2>	4.14	60,018	13.7	7,168	25.3
23_MOZ	Jasbrouk	4.16	61,093	19.1	8,929	27.0
24_MYA	Miandianhei	4.19	253,189	23.0	44,538	27.5
25_PHI	CLSU-1	4.08	200,737	22.3	16,553	27.9
26_UAE	24-1	4.12	260,055	23.1	31,722	27.2
27_USA	u.C.R/82NOINS	4.16	54,410	21.3	5,364	27.9
28_USA	Youxianxing N03	4.18	215,692	31.9	26,637	32.7
29_VIE	V6	4.17	40,925	15.8	5,363	20.3

Although sesame is traditionally considered a self-pollinating plant, it is also subjected to crossed pollination by insects such as butterflies and bees. This is consistent with the observed rate of heterozygosity ranging from 12.37 to 49.30%, with an average of 25.39% (Additional file 2: Figure S6; Additional file 1: Data S5). Five of the 16 Chinese cultivars (strains 1 to 5) exhibited lower heterozygous rates (16.82 - 23.25%) compared to both mean levels and other non-Chinese strains. The unusually high heterozygosity rates for strains 16, 22, and 26 suggest that these strains are more attractive to insects; however, more trivial explanations, such as sequencing and alignment errors, cannot be ruled out, especially in the repeat regions.

**Figure 2:**
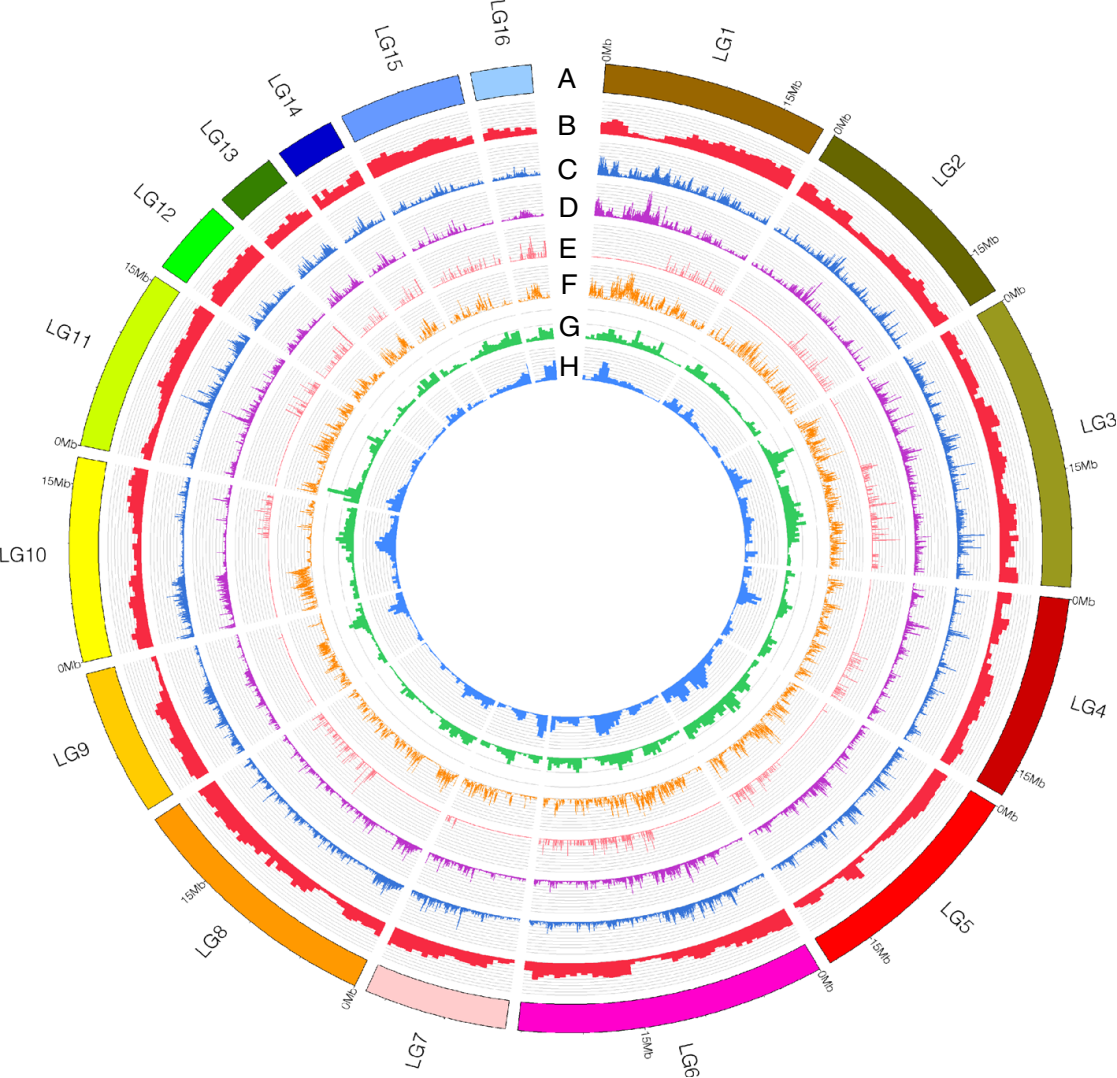
**Landscape of the genetic variation in sesame.** Distribution of **(A)** pseudomolecules, **(B)** gene density (mRNA), **(C)** average InDel density, **(D)** population SNPs, **(E)** large-effect SNPs, **(F)** π values, **(G)** DNA transposon element density, and **(H)** retrotransposon element density across the sesame genome.

Population SNPs were used to calculate two commonly used population genetics statistics: population diversity (*π*) and Watterson’s estimator of segregating sites (*θw*). The average *π* and *θw* values across all 29 strains were 2.5 and 3.0 per kb, respectively, which are lower than that of rice [12] but higher than chickpea (*Cicer arietinum*) [13], watermelon (*Citrullus lanatus*) [14] and soybean [15] (Additional file 1: Data S6). We observed numerous consecutive slide windows along with the pseudomolecules (LG1-LG16) that contained fewer than normal SNPs, and in turn lower *π* and *θw* values (Figure 2), indicative of an uneven distribution of genetic diversity along sesame pseudomolecules. We examined the genome for the highest and lowest polymorphic regions (blocks falling in the top and bottom 5% of *π* values) and found that the number of genes in the highest polymorphic regions was smaller than in the lowest regions (524 vs. 1308) (Additional file 1: Data S7 and S8), similar to other species, such as chickpea [13]. Many of the genes in the highest polymorphic regions were related to environmental adaptability, including stress response pathways (Additional file 1: Data S9). These genes may offer a valuable resource for the study of biotic and abiotic stress in sesame. It is also interesting to note that despite the greater number of genes in the lowest polymorphic regions, only five genes were enriched in two gene ontology (GO) terms, all of which were associated with basic biological functions; i.e., ribosome binding (Additional file 1: Data S10).

We next used the mapped reads that met all pair-end requirements, but contained alignment gaps in one end of the contig to detect short InDels (1 - 5 bp) in each strain. The overall number of InDels detected was inversely proportional to the length of the InDel (Additional file 2: Figure S7). The numbers identified across all 29 strains ranged from 4,495 - 56,594 (average = 17,961), for a total of 520,880 unique InDels (Additional file 1: Data S11). Similar to SNPs, the distribution of InDels along the genome was not uniform, with high-density regions strongly associated with regions containing high SNP density (Figure 2). Among these InDels, the numbers of insertions and deletions were similar (48.8% vs. 51.2%, respectively). Homozygous InDels were found at a rate more than 1.5-fold that of heterozygous InDels. Of these InDels, 71.7% were located in intergenic regions, 1.5% (8,221) in CDS and 5.0% in UTRs, respectively.

Structural variation (SV) was originally defined as insertions, deletions, DNA inversions and other sequence rearrangements greater than 1 kb in size [16]. With sequencing now becoming routine [17], the operational spectrum of structural variants has widened to include much smaller events [18,19]. In the present study, we detected SVs between 10 bp and 1 Mb using the software package Breakdancer v1.2 [20] set to default parameters. We found 7,220 - 12,458 SVs per strain (average = 9,266) across all 29 strains when compared to the reference genome (Additional file 1: Data S12). For these SVs, deletion events outnumbered insertions at a rate of nearly two to one (Additional file 2: Figure S8). Outside of InDels, the rates of other SVs, including DNA inversion, intrachromosomal translocation and interchromosomal translocation were relatively low, ranging from 739 to 2,360 (average = 1,140). The majority of SVs were between 100 - 1000 bp in size, with longer variations (>1 kb) less abundant, especially those longer than 10 kb (Additional file 2: Figure S9), consistent with that seen in sorghum [21].

### The effect of variations on genes

DNA sequence changes within genes plays a pivotal role in morphology and plant evolution. Of the 27,148 annotated genes in sesame genome (http://www.ocri-genomics.org/Sinbase/), 74.8% (20,311) were found to contain one or more SNPs in comparison to the reference genome. Furthermore, 62.6% (16,997), 15.5% (4,218), and 18.0% (4,892) of genes contained SNPs in their CDS, 5′UTRs, and 3′UTRs, respectively. These genes were categorized into 43 molecular function groups, with 30% associated with the terms protein binding, hydrolase activity and ATP binding; however, all genes with predicted hydrolase activity contained SNPs only within their CDS regions (Additional file 2: Figure S10). Further analysis identified 258 genes with SNPs in their CDS regions that were significantly enriched (*P* < 0.01) for the biological processes cell death and apoptotic process (Additional file 1: Data S13). The 136,130 non-synonymous and 142,103 synonymous SNPs identified in coding regions represent a non-synonymous-to-synonymous substitution ratio of 0.99 (Additional file 1: Data S4; Additional file 2: Figure S11), similar to that of sorghum (1.0) [22], but higher than that of *Arabidopsis thaliana* (0.83) [23] and lower than that of soybean (1.38) [15] and rice (1.2) [24]. GO term enrichment for genes with non-synonymous SNPs were strongly associated with cell death, apoptosis, and defense response (Additional file 1: Data S14), particularly those genes encoding disease resistance proteins, UDP-glucosyltransferase or the proteins containing leucine-rich repeats and NB-ARC domains (Additional file 2: Figure S12; Additional file 1: Data S15). These results are indicative of a higher rate of mutation in genes involved in biotic stress responses, consistent with the theory that plant-pathogen interactions result in the diversification of pathogen-associated molecular pattern recognition receptors [25,26].

Coding region SNPs located in key structural locations can lead to significant changes in protein morphology, and in turn cause changes in overall protein function. Within the 29 sesame strains examined, we identified 1,281 SNPs associated with the formation of premature stop codons and 246 stop codon to non-stop codon mutations. Start codon to non-start codon mutations were observed in 186 genes, along with an additional 404 splice site mutations (Additional file 2: Figure S13). Most of these large-effect SNPs were located on the proximal ends of the pseudomolecules (LG) (Figure 2). Annotation of these four large-effect SNPs categories revealed different patterns of functional enrichment. For example, start codon to non-start codon mutations were found primarily in genes involved in transport, apoptosis, and defense response, while splice site mutations were more common in genes associated with cellular metabolism, oxidation-reduction, organic substance metabolism, and nitrogen compound metabolism (Additional file 2: Figure S14). Among the four types of large-effect SNPs, premature stop codons were particularly interesting, as these mutations are often associated with loss of function. The majority of the mutations were found in genes associated with the GO biological processes related to adversity, including cell death, apoptosis, and defense response (Additional file 1: Data S16).

Despite the fact that most SNPs were detected in CDS regions, CDS regions accounted for only 14.3% of the 12,651 InDel mutations, lower than both the 5′ and 3′UTRs (18.4% and 19.6%, respectively). The number of genes containing InDels in the 5′ and 3′ UTRs decreased as InDel size increased from 1 to 5 bp, but the CDS InDels increased significantly in 3-bp InDels, similar to that observed in sorghum [21] (Additional file 2: Figure S15). This enrichment of 3-bp InDels is to be expected, as InDels that are not multiples of 3 bp result in frame shifts and are usually fatal. Finally, we analyzed the distribution of InDels on the basis of gene ontology, and found they were similar to SNPs resulting in premature stop codons, with statistically significant enrichment (*P* < 0.001) in genes involved in cell death, apoptosis, and defense response (Additional file 1: Data S17).

### Genetic relationships among the 29 sesame strains tested

**Figure 3:**
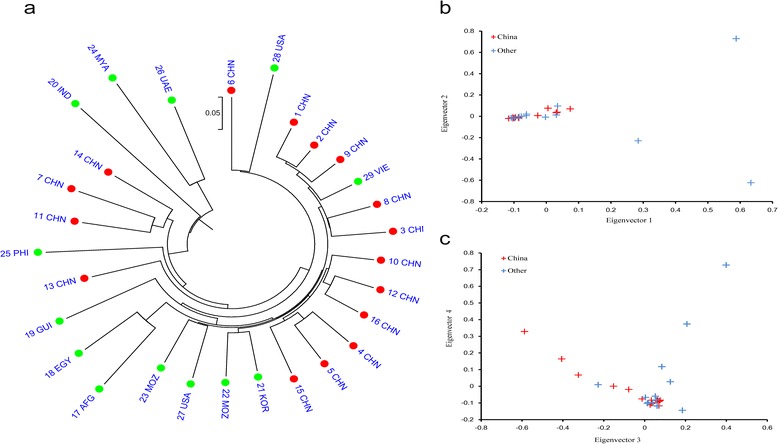
**Genetic relationships among the 29 sesame strains tested. (a)** Neighbor-joining (NJ) tree analysis of 29 sesame strains based on population SNPs. **(b, c)** PCA results for the first four statistically significant components.

**Figure 4:**
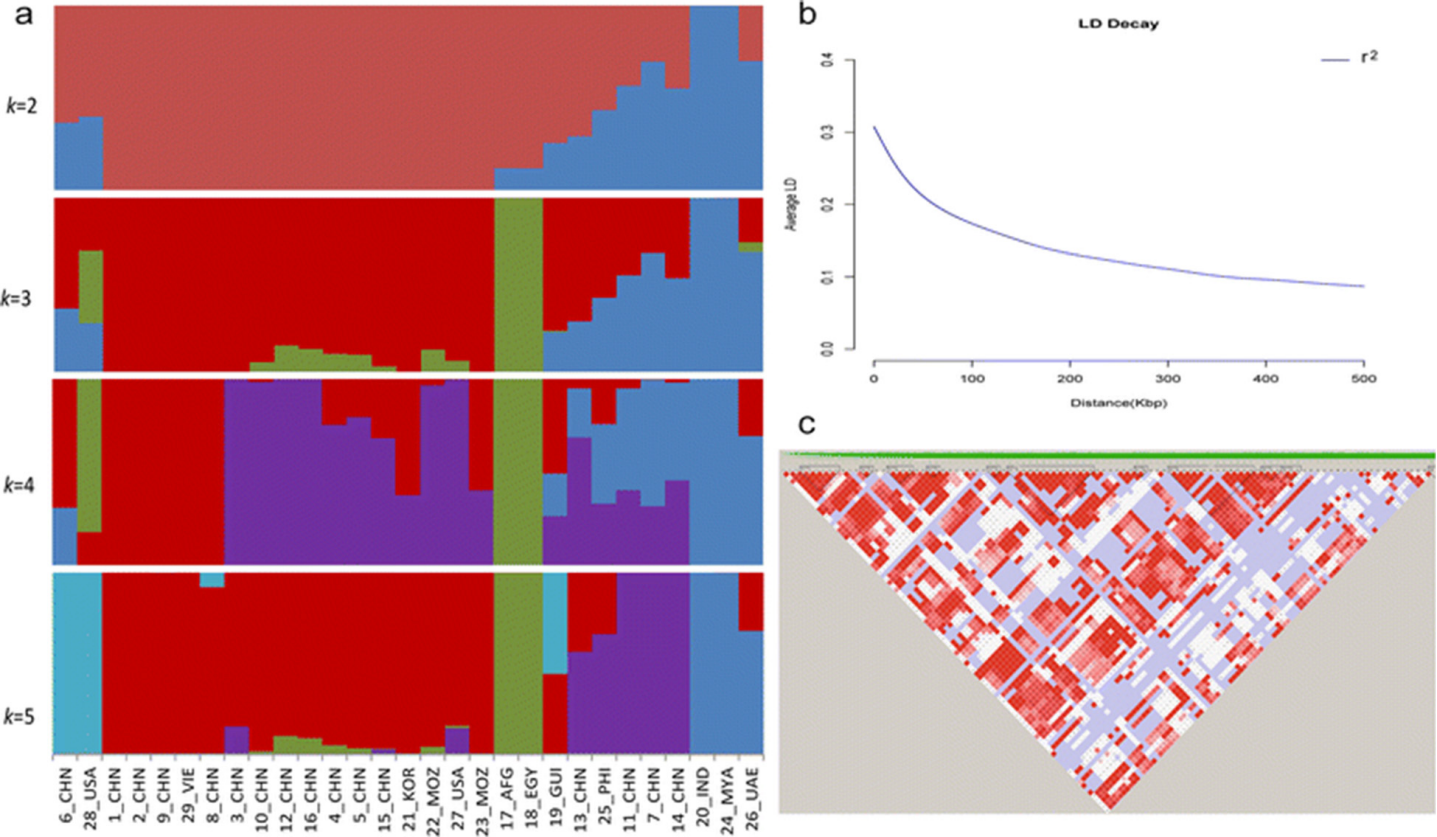
**Population structure and linkage disequilibrium of sesame. (a)** Structure analysis of 29 sesame strains based on whole-genome sequencing. **(b)** LD decay determined using squared correlations of allele frequencies (r^2^) against distance in sesame. **(c)** LD of sesame, shown using a slide window of 100 kb. Red and white spots indicate strong (r^2^ = 1) and weak (r^2^ = 0) LD, respectively.

When considering whether sesame was first cultivated in Africa or on the Indian subcontinent [8,9], it is important to investigate the effects of geography on sesame genetic diversity. A phylogenetic tree containing all 29 sesame strains was constructed using the neighbor-joining method. This analysis revealed the highest degrees of relatedness among the Chinese strains, with strains originating in other countries spread throughout (Figure 3a). This interwoven nature of sesame strains derived from different geographic locations was also evident based on principal component analysis (PCA) (Figure 3b and c). Indistinct groups were observed using the Bayesian clustering software STRUCTURE [27] with K changing progressively from 2 – 5 (Figure 4a).

As this study did not include any relatives or wild species of sesame, definitive conclusions regarding the origins of sesame are not possible. However, the phylogenetic relationships observed among the 29 sesame strains shed some light on the evolution of sesame. The three strains from India, Myanmar, and the United Arab Emirates exhibited higher genetic distances relative to the other strains (Figure 3a). According to the Vavilov center of diversity theory, which states that richer genetic diversity is observed in the location where a plant was first domesticated [28], these results suggest that sesame originated on the Indian subcontinent.

### High linkage disequilibrium in sesame

LD patterns are necessary to determine mapping resolution when designing association studies [29,30] and interpreting association peaks [31]. To estimate the LD of sesame, we calculated r^2^ between pairs of SNPs using Haploview [32] and found that it decayed to ~0.15 from an initial value of 0.30 over the course of ~150 kb (Figure 4b and c). The LD decay estimate of sesame is comparable to that of self-pollinated soybean (~150 kb) [15], but higher than that seen in *A. thaliana* (~4 kb) [29], indica rice (~65 kb) [12] and foxtail millet (~100 kb). It was also significantly higher than that of cross-pollinated plants such as sorghum (1 kb) [33] and maize (<1 kb) [34]. The high LD of sesame makes it not only a good plant for studying the effects of extreme LD in genomic and population structures [15], but also suitable for GWAS with relatively few polymorphic markers.

### Bulked segregant analysis for the candidate sites of the number of flowers per leaf axil in sesame

**Figure 5:**
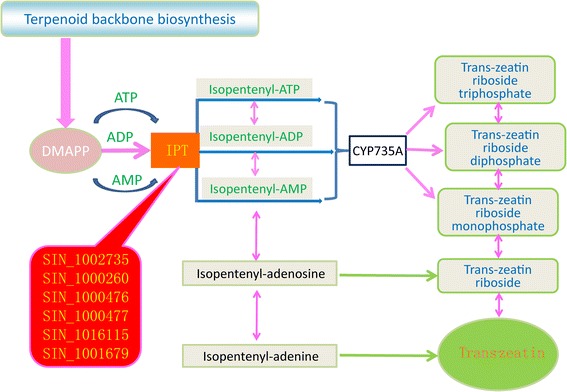
**Positions of the six ITP homologs in the zeatin biosynthesis pathway.** DMAPP: Dimethylallyl pyrophosphate; CY735A: Cytokinin trans-hydroxylase; ATP: Adenosine triphosphate; ADP: Adenosine diphosphate; AMP: Adenosine monophosphate.

Bulked segregant analysis (BSA) is a rapid method that allows for the detection of markers in specific genomic regions [35] and has been successfully applied to detect quantitative trait loci (QTL) or genes for various traits in rice [36], maize [37], and wheat [38]. In combination with high-throughput sequencing technology, BSA has been used to identify a novel xylose utilization gene from *Saccharomyces cerevisiae*. Here, we employed the BSA method to explore candidate genes that may be responsible for the number of flowers per leaf axil. This phenotype is an important agronomic trait in sesame as it plays a role in the predicted yield. The 29 sesame strains were classified into two groups based on mono-flower versus triple-flower (13 versus 16) (Additional file 1: Data S1). We identified 695 genes with coincident SNPs between the two pools. Of these genes, 181, 21 and 31 contained SNPs in the CDS, 5′UTR and 3′UTR, respectively (Additional file 1: Data S18). GO term annotation associated these genes predominantly with ATP binding, zinc ion binding, nucleic acid binding and heat shock protein binding. Of particular interest were six adenylate isopentenyltransferase (ITP) homologs (SIN_1002735; SIN_1000260; SIN_1000476; SIN_1000477; SIN_1016115 and SIN_1001679), which were significantly enriched in the zeatin biosynthesis pathway (Figure 5). Zeatin is a member of the phytohormone family of cytokinins, which is known to be involved in a variety of processes associated with the growth and development of plants, including promotion of lateral bud growth and stimulation of cell division to produce bushier plants [39,40]. The present results suggest that ITP genes may also play a role in the number of flowers per leaf axil of sesame by mediating zeatin biosynthesis. However, further studies using transgenic models or two-parent crossing populations are required.

## Conclusions

Next-generation sequencing is rapidly increasing our understanding of genetic variation in crop plants [41]. This study provides the first comprehensive resequencing analysis of the high oil crop sesame. The availability of these data, generated from 29 strains originating from 12 countries, provides insight into genetic variation of the sesame germplasm genome and facilitates a broad range of functional and evolutionary studies including on genome evolution, population genetics, marker-assisted breeding and gene identification. The identification of high LD in the sesame genome indicates that marker-assisted breeding is a better choice for sesame improvement. The data presented here provide new evidence supporting the hypothesis that sesame originated on the Indian subcontinent. In both coding and noncoding regions, we identified hundreds of thousands of polymorphisms, which provide an important resource for both evolutionary genetic and functional studies. Of particular interest are genes harboring non-synonymous mutations, including large-effect SNPs, which are likely to mediate interactions with the environment. This study also suggested that the ITP genes might play a role in determining the number of flowers per leaf axil of sesame. However, further studies are required to fully understand the functional relevance of the genetic variations identified in this study.

## Methods

Twenty-nine cultivated sesame strains were selected for genome resequencing, including 16 from China, 2 from the United States, and 1 each from Afghanistan, the United Arab Emirates, Korea, Myanmar, the Philippines, and Viet Nam.

### Library construction and sequencing

Genomic DNA was extracted from fresh and etiolated leaves of each strain using the CTAB method. Paired-end sequencing libraries with inset sizes of ~500 bp were constructed for each strain according to the manufacturer’s instructions (Illumina) using 5-μg genomic DNA. Sequencing was performed using the Illumina Hiseq 2000 platform. Raw sequencing reads were then subjected to a series of stringent filtering steps, removing reads based upon the following criteria:

Type (1): Reads with ≥ 10% and ≥ 3% unidentified nucleotides for short and long insert size libraries, respectively.

Type (2): Reads having > 40% of bases with quality scores < 7.

Type (3): Reads of > 10 bp aligned to the adapter sequence, allowing ≤ 2-bp mismatches.

Type (4): Paired-end reads that overlapped ≥ 10 bp with the corresponding paired end.

Type (5): Read1 and read2 of two paired-end reads that were completely identical (considered to be products of PCR duplication).

A total of > 120 Gb was generated following all filtering steps, at a depth of ≥ 13-fold (Additional file 1: Data S1).

### SNP calling

Reads were mapped to the assembled sesame genome of “Zhongzhi No.13” using BWA software [10]. The detailed parameters used were as follows:

“bwa aln -m 200000 -o 1 -e 30 -i 15 -l 35-L -I -t 4 -n 0.04 -R 20 –f”

“bwa sampe -a 800”

Considering all strains as a group, we used the SAMtools function “mpileup” [11] to detect raw population SNPs using reads with a mapping quality ≥ 20. The parameters used were as follows:

“samtools mpileup –uf –b –D| bcftools view -bvcgI -p 0.99”

Using the SAMtools program “vcfutils”, SNPs extracted using the above process were first filtered to yield sequencing depths between 30 and 581. The parameters used were as follows:

“perl vcfutils.pl varFilter -d 30 -D 581”

Raw SNP sites were further filtered based on the following criteria: copy number ≤ 2, and a minimum of 5 bp apart, with the exception of minor allele frequencies (MAF ≥ 0.05) where SNPs were retained when the distance between SNPs was < 5 bp. The diversity parameters *π* and *θ*_*w*_ were measured using a window of 10 kb with a sliding window of 1 kb [12,14].

To check the SNP calling accuracy of SAMtools, four fragments ranging in size from 4.5 to 8.1 kb were randomly selected and amplified using overlapped primers, and the resulting PCR products subjected to Sanger sequencing. The concordance rates of SNPs detected by the two methods ranged from 92.3 to 95.2 (average = 93.7%) (Additional file 2: Figure S4; Additional file 1: Data S3).

In addition, the GATK toolkit [42] was also used to call SNPs, as follows:

We first mapped clean reads to the sesame genome using the BWA software with the following parameters:

“bwa aln -m 200000 -o 1 -e 30 -i 15 -l 35-L -I -t 4 -n 0.04 -R 20 –f”

“bwa sampe -a 800”.

SAMtools was used to split, sort, rmdup and merge the SAM aligned result, and picard-tools was used to sort the bam result and was marked as the duplicate. Next, we used the GATK program to realign and filter SNPs from the unified genotyper raw VCF using the parameters:

java -jar GenomeAnalysisTK.jar -T SelectVariants –R –variant –concordance –o

java –jar GenomeAnalysisTK.jar -T VariantFiltration –R --filterExpression “QD < 20.0 || ReadPosRankSum < -8.0 || FS > 10.0 || QUAL < $MEANQUAL” --filterName LowQualFilter --missingValuesInExpressionsShouldEvaluatAsFailing –variant --logging_level ERROR –o

java –jar GenomeAnalysisTK.jar -T CombineVariants –R -V sample1.vcf –V sample2.vcf -genotypeMergeOptions UNIQUIFY –o *.

A total of 2,003,821 population SNPs were obtained from the 16 linkage groups using GATK.

### Short InDel detection

Using the default parameters of the software SOAPInDel [43], primary short insertions or deletions up to 5 bp were extracted based on the mapped reads that meet the pair-end requirements and contain alignment gaps, with all gaps supported by at least three non-redundant paired-end reads. Primary InDel sets were then filtered to include read quality values > 20 and InDels < 5 bp away.

### Structure variation detection

According to the principal of paired-end sequencing, one of the paired-end reads should be aligned to the forward sequence, while the other is aligned to the reverse sequence. The distance between the two aligned positions should be in accordance with the insert size. Thus two paired-end reads aligned to the genome should have normal direction and appropriate span. Abnormal paired-end alignments were analyzed by clustering and comparing with the types of structure variation previously defined using the software Breakdancer [20] run using the default parameters. The resulting SV dataset included INS (insertions), DEL (deletions), ITX (intrachromosomal translocations), INV (inversions) and CTX (interchromosomal translocations) ranging from 10 bp to 1 Mb.

### Calculation of linkage disequilibrium

To measure LD in the population, we calculated the correlation coefficient (r^2^) of alleles using the software Haploview [32], as follows:

1. Ped and info files were generated as input files.
2. For each chromosome, such as LG1, the parameters were set as “java –jar haploview.jar -n –log LG1.log -pedfile LG1.genotype.ped -info LG1.genotype.info -dprime -minGeno 0.6 -minMAF 0.01 -hwcutoff 0.001 -memory 2000 -maxdistance 500”.
3. Curves were then plotted with R scripts, which draw averaged (r^2^) against pair wise marker distances.

### Population genetics analysis

The diversity parameters *π* and *θ*_*w*_ were measured using a window of 10 kb with a sliding window of 1 kb [12,14]. The top and bottom 5% blocks based upon *π* value were extracted, and the genes in these blocks defined as high- and low-divergence genes, respectively (Additional file 1: Data S7 and S8).

Individual SNPs were used to calculate distances between samples. Under the p-distances model with bootstrapping (1,000), a neighbor-joining tree was constructed with TreeBest (http://sourceforge.net/projects/treesoft/files/treebest/) for the 29 sesame strains. The phylogenetic tree was displayed using the software MEGA5 [44]. was performed using the software EIGENSOFT [45]. The software FRAPPE [46] was used to determine the population structure.

## Additional files

Additional file 1:
**Consists of the supplementary Data S1 to S18.** **Data S1.** Information on the 29 resequenced sesame strains. **Data S2.** Comparison of the calling SNPs between GATK and Samtools. **Data S3.** Validation of the SNPs detected with NGS using Sanger sequencing in four DNA fragments. **Data S4.** Number of SNPs in gene regions. **Data S5.** Summary of the candidate SNPs in the 29 strains. **Data S6.** Diversity levels of sesame and other species populations. **Data S7.** List of genes in the blocks with the highest π value (top 5%). **Data S8.** List of genes in the blocks with the lowest π value (top 5%). **Data S9.** Enriched GO terms of genes in regions with the highest π (top 5%). **Data S10.** Enriched GO terms of genes in regions with the lowest π (top 5%). **Data S11.** Summary of InDels detected in the 29 strains. **Data S12.** Summary of SVs detected in the 29 strains. **Data S13.** Enriched GO terms for genes containing SNPs in the CDS. **Data S14.** Enriched GO terms for genes containing non-synonymous SNPs in the CDS. **Data S15.** Enriched IPR terms for genes containing non-synonymous SNPs in the CDS. **Data S16.** Enriched GO terms for genes containing large-effect SNPs. **Data S17.** Enriched GO terms for genes containing frameshift InDels in the CDS. **Data S18.** Numbers of mutant genes related to various phenotypes predicted using BSA. Additional file 2:
**Consists of the supplementary Figure S1 to S15.** **Figure S1.** Clean data of the 29 sesame strains acquired using next-generation sequencing technology. **Figure S2.** Numbers of total SNPs and SNPs located in mRNA regions in the 29 sesame strains. **Figure S3.** Comparison of the frequencies of different SNP styles in sesame. **Figure S4.** Chromas exemplifying the SNPs discordant between NGS and Sanger sequencing. **Figure S5.** Statistics of the SNPs located in the UTR and CDS in the 29 sesame strains. **Figure S6.** Proportions of the heterozygous and homozygous SNPs in the 29 sesame strains. **Figure S7.** Number of InDels of 1 to 5 bp in the 29 sesame strains. **Figure S8.** Number of SVs in each of the 29 sesame strains. **Figure S9.** Length distributions of SVs in the 29 sesame strains. **Figure S10.** GO-SLIM categories of the genes with SNPs in the CDS, 5′-UTR and 3′-UTR. **Figure S11.** Proportions of synonymous and non-synonymous SNPs in CDS regions. **Figure S12.** Proportions of genes containing non-synonymous SNPs in different gene families. **Figure S13.** Proportions of genes containing large-effect SNPs. **Figure S14.** GO-SLIM categories of genes containing large-effect SNPs. **Figure S15.** Numbers of genes with different InDels in the CDS, 5′-UTR, and 3′-UTR.

## Abbreviations

BSA: Bulked segregant analysis
CDS: Coding sequence
CTX: Interchromosomal translocation
InDel: Insertion and deletion
GO: Gene ontology
GWAS: Genome-wide association study
INS: Insertion
ITX: Intrachromosomal translocation
INV: Inversion
ITP: Isopentenyltransferase
LD: Linkage disequilibrium
PCA: Principal component analysis
RDA: Rural development administration
SNP: Single nucleotide polymorphism
SV: Structure variation
UTR: Untranslated region
