# Supplementary material for: Deep resequencing reveals allelic variation in Sesamum indicum
Source: BMC Plant Biol. 2014 Aug 20;14:225. doi: 10.1186/s12870-014-0225-3 (PMC4148021; doi:10.1186/s12870-014-0225-3)
Supplement: Additional file 2: — Consists of the supplementary Figure S1 to S15. Figure S1. Clean data of the 29 sesame strains acquired using next-generation sequencing technology. Figure S2. Numbers of total SNPs and SNPs located in mRNA regions in the 29 sesame strains. Figure S3. Comparison of the frequencies of different SNP styles in sesame. Figure S4. Chromas exemplifying the SNPs discordant between NGS and Sanger sequencing. Figure S5. Statistics of the SNPs located in the UTR and CDS in the 29 sesame strains. Figure S6. Proportions of the heterozygous and homozygous SNPs in the 29 sesame strains. Figure S7. Number of InDels of 1 to 5 bp in the 29 sesame strains. Figure S8. Number of SVs in each of the 29 sesame strains. Figure S9. Length distributions of SVs in the 29 sesame strains. Figure S10. GO-SLIM categories of the genes with SNPs in the CDS, 5′-UTR and 3′-UTR. Figure S11. Proportions of synonymous and non-synonymous SNPs in CDS regions. Figure S12. Proportions of genes containing non-synonymous SNPs in different gene families. Figure S13. Proportions of genes containing large-effect SNPs. Figure S14. GO-SLIM categories of genes containing large-effect SNPs. Figure S15. Numbers of genes with different InDels in the CDS, 5′-UTR, and 3′-UTR. [file 12870_2014_225_MOESM2_ESM.docx]

**Figure S1** Clean data of the 29 sesame strains acquired using next-generation sequencing technology

**Figure S2** Numbers of total SNPs and SNPs located in mRNA regions in the 29 sesame strains


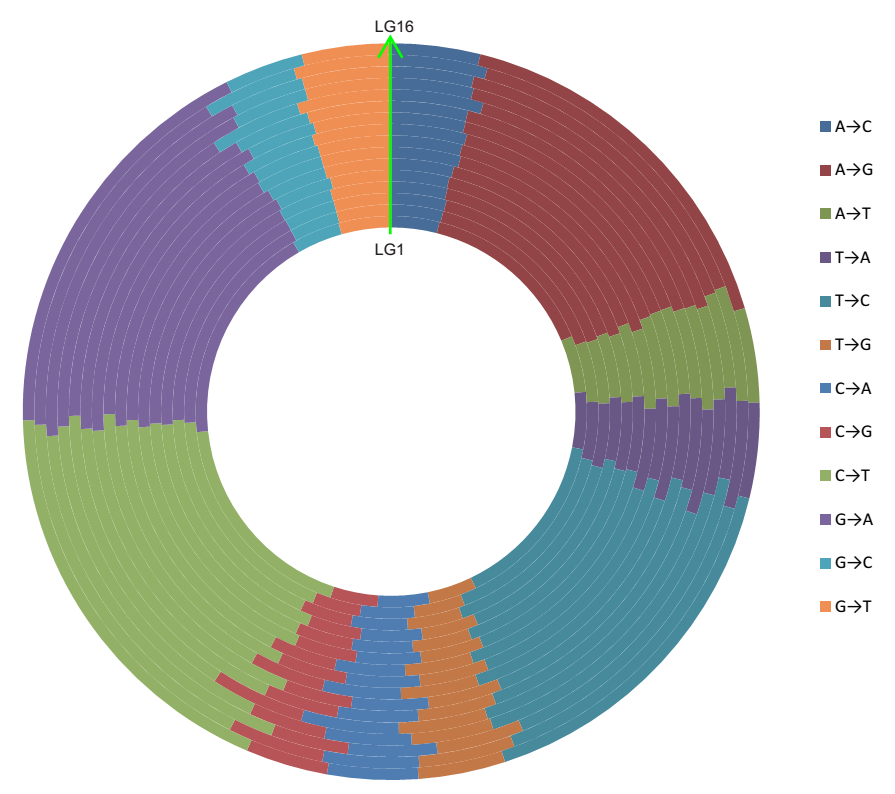


**Figure S3** Comparison of the frequencies of different SNP styles in sesame


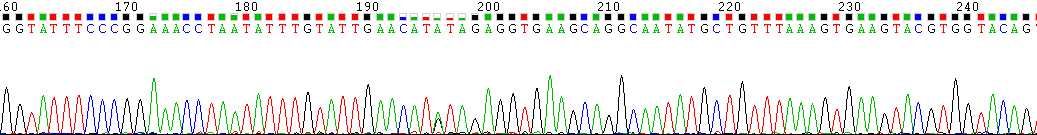


**a**


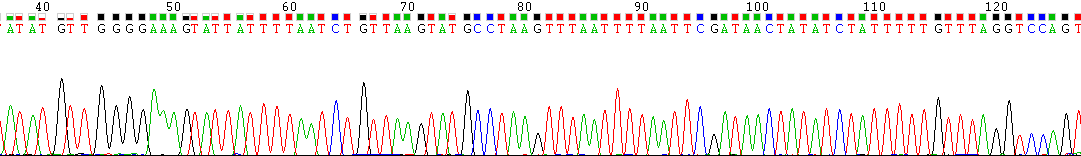


**b**

**Figure S4** Chromas exemplifying the SNPs discordant between NGS and Sanger sequencing. a: In the Sample 15_CHN, a new SNP T\C (Y) at the locus 15,239,637 of LG11 was found by Sanger (Reverse and complement sequencing result of Sanger was illustrated); b: In the Sample 20_IND, the SNP at the locus 15,240,815 of LG11 was detected by NGS (G to A), but not get by Sanger.

**Figure S**5 Statistics of the SNPs located in the UTR and CDS in the 29 sesame strains

**Figure S6** Proportions of the heterozygous and homozygous SNPs in the 29 sesame strains

**Figure S7** Number of InDels of 1 to 5 bp in the 29 sesame strains

**Figure S8** Number of SVs in each of the 29 sesame strains

**Figure S9** Length distributions of SVs in the 29 sesame strains

**a**

**b**

**c**

**Figure S10** GO-SLIM categories of the genes with SNPs in the CDS, 5’-UTR and 3’-UTR. (a) Biological processes; (b) Cellular Components; (c) Molecular Function

**Figure S**11 Proportions of synonymous and non-synonymous SNPs in CDS regions

**Figure S**12 Proportions of genes containing non-synonymous SNPs in different gene families

**Figure S**13 Proportions of genes containing large-effect SNPs

**a**

b

**c**

**Figure S14** GO-SLIM categories of genes containing large-effect SNPs. (a) Biological processes; (b) Cellular Components; (c) Molecular Function

**Figure S15** Numbers of genes with different InDels in the CDS, 5’-UTR, and 3’-UTR. D1: Deletions of 1 bp; I1: Insertions of 1 bp.
